# Accurate Correlation Modeling between Wind Speed and Bridge Girder Displacement Based on a Multi-Rate Fusion Method

**DOI:** 10.3390/s21061967

**Published:** 2021-03-11

**Authors:** Yan Wang, Dong-Hui Yang, Ting-Hua Yi

**Affiliations:** School of Civil Engineering, Dalian University of Technology, Dalian 116023, China; wy89@mail.dlut.edu.cn (Y.W.); dhyang@dlut.edu.cn (D.-H.Y.)

**Keywords:** multi-rate Kalman fusion, wind speed-displacement modeling, structural health monitoring, cable-stayed bridge, performance warning

## Abstract

Wind action is one of the environmental actions that has significant static and dynamic effects on long-span bridges. The lateral wind speed is the main factor affecting the lateral displacement of the main girder of the bridge. The main objective of the paper is to use the improved multi-rate fusion method to correct the monitoring data so that accurate correlation modeling of wind speed-displacement can be achieved. Two Kalman gain coefficients are introduced to improve the traditional multi-rate fusion method. The fusion method is verified by the results of simulated data analysis in time domain and frequency domain. Then, the improved multi-rate fusion method is used to fuse the monitoring lateral displacement and acceleration data of a bridge under strong wind action. The corrected lateral wind speed and displacement data is further applied to establish the correlation model through the linear regression. The improved multi-rate fusion method can overcome the inaccuracy of the high frequency stage of a Global Positioning System (GPS) sensor and the low frequency stage of acceleration sensor. The correlation coefficient of wind speed-displacement after fusion increases and the confidence interval width of regression model decreases, which indicates that the accuracy of the correlation model between wind speed and displacement is improved.

## 1. Introduction

Recently, more and more large civil buildings and long-span bridges are being constructed [1]. These structures and bridges will inevitably be damaged in the long-term service period, and their performance will deteriorate due to material fatigue, environmental corrosion, and environmental load [2,3]. Bridge monitoring data includes structural monitoring data, environmental monitoring data, etc. Direct correlation analysis of these monitoring data can realize damage identification performance for early-warning of bridge structures, which helps to monitor the performance of bridges and prevent catastrophic accidents through security warnings [4,5]. Structural health monitoring systems using sensors can monitor the deformation of bridges [6,7,8]. Damage identification of bridges can be carried out through big data analytics of a bridge monitoring system [9]. A structural health monitoring system can establish big data to extend the service life of bridges [10,11].

This paper aims to improve the modeling accuracy of lateral wind speed and displacement in bridge mid-span. Monitoring data of the bridge have the advantages of being continuous and long-term, which can realize early warning of the structural damage [12,13]. A multivariate statistical analysis method was used to eliminate the nonlinear aeroelastic correlation between dynamic characteristics and wind speed and to monitor the main cable of a suspension bridge [14]. Fenerci et al. studied the relationship between wind load and responses of a suspension bridge based on monitoring data, and the wind parameters that were analyzed [15]. Early warning of the structural damage can be realized by modeling the correlation between structural monitoring data and environmental monitoring data. Previous studies have shown that there is a direct correlation between temperature and displacement, and the correlation modeling can be used to realize the bridge performance warning [16,17]. The influence of wind on bridge structure can be obtained by statistics of average wind speed, wind direction, wind turbulence intensity, integral scale, etc. [18]. The influence of strong wind on bridge displacement can be known by studying the monitoring data [19,20]. Ye presents the construction of the bivariate model of wind speed and direction of an arch bridge by use of the long-term structural health monitoring (SHM) data [21,22].

The multi-rate fusion method can modify the monitoring data so as to achieve the correlation model of wind speed and displacement data. The fusion method is widely used in the field of building structure and long-span bridge structure, which can be divided into three types. Firstly, the fusion method is used for damage identification of civil buildings, bridge structures, etc. Damage identification of large span bridges is carried out by means of identifying influence lines and fusion of multiple influence lines [23,24,25]. Secondly, the fusion method is utilized to reconstruct unknown data through existing data. The data of some monitoring points are difficult to measure or arrange sensors can be reconstructed by means of fusion with the data of known monitoring points [26,27,28]. Thirdly, the fusion method is used for fusing displacement and acceleration data fusion, which utilize the accuracy of the displacement data in the low frequency stage and the acceleration in the high frequency stage to modify both data simultaneously [29,30]. Chang et al. used the fusion method to fuse the displacement and acceleration measured in accordance with the camera, and the results also demonstrated the effectiveness of the method [31]. In order to apply the fusion method to bridge monitoring data, the existing fusion method needs to be improved. Errors after correlation modeling can be used for bridge performance warning. Huang used the monitoring data to detect potential performance degradation of bridges [32,33].

Accurate data is important for a correlation model of wind speed and displacement data, but the monitoring data is affected by noise and so on. In addition, the existing fusion method cannot modify the displacement and acceleration monitoring data simultaneously. In this paper, the authors proposed an improved multi-rate fusion method, and the correlation modeling of wind speed and displacement of large span cable-stayed bridge under strong wind action is studied. The paper is organized as follows: first, the multi-rate Kalman fusion method is introduced. The multi-rate fusion method is improved by setting two Kalman gain coefficients, which is used to fuse the simulated data. Second, the monitoring data of a bridge were studied and data under strong wind action is selected as cases 1 to 4. Then, the improved multi-rate fusion method is used to fuse the monitoring data, and the changes of the time domain and frequency domain are considered. Third, the correlation analysis and the regression model were built by using fused wind speed and displacement monitoring data, which is compared with the model before fusion. Fourth, the effectiveness of the fusion method is verified by the Shewhart control chart in the bridge performance warning research. Finally, some detailed conclusions are presented.

## 2. Improved Kalman Multi-Rate Fusion Method

In this paper, the multi-rate fusion method is used to modify the displacement and acceleration data with different sampling rates. This paper improves the traditional multi-rate fusion method by setting two Kalman gain coefficients so that the improved multi-rate fusion method can better modify the bridge monitoring data. The displacement and acceleration monitoring data can be corrected simultaneously by using the improved multi-rate fusion method.

### 2.1. Traditional Multi-Rate Fusion Method

Consider the case that acceleration and displacement can be measured. Then, the measurement process is modeled in equation form as:

(1) $$\left[\begin{array}{l} \dot{x}\\ \ddot{x} \end{array}\right]=\left[\begin{array}{cc} 0 & 1\\ 0 & 0 \end{array}\right]\left[\begin{array}{l} x\\ \dot{x} \end{array}\right]+\left[\begin{array}{l} 0\\ 1 \end{array}\right]{\ddot{x}}_{a}+\left[\begin{array}{l} 0\\ 1 \end{array}\right]z_{c}$$

(2) $$\left[x_{d}\right]=\left[\begin{array}{cc} 1 & 0 \end{array}\right]\left[\begin{array}{l} x\\ \dot{x} \end{array}\right]+\left[z_{d}\right]$$

where $\dot{x}$ and $\ddot{x}$ are the fused velocity and displacement, $z_{d}$ and $z_{c}$ are the associated measurement noise of acceleration and displacement, and $x_{d}$ and ${\ddot{x}}_{a}$ are the measured displacement and acceleration. It is assumed that $z_{d}$ and w are white noise Gaussian processes.

### 2.2. Improved Multi-Rate Fusion Method

In order to better combine with the measured data of the bridge, the traditional multi-rate fusion is improved in this paper. The measurement equation of acceleration and displacement can be expressed as:

(3) $$\left[\begin{array}{l} \dot{x}\\ \ddot{x}\\ \dddot{x} \end{array}\right]=\left[\begin{array}{ccc} 0 & 1 & 0\\ 0 & 0 & 1\\ 0 & 0 & 0 \end{array}\right]\left[\begin{array}{l} x\\ \dot{x}\\ \ddot{x} \end{array}\right]+\left[\begin{array}{l} 0\\ 0\\ 1 \end{array}\right]z_{c}$$

(4) $$\left[\begin{array}{l} x_{d}\\ {\ddot{x}}_{a} \end{array}\right]=\left[\begin{array}{ccc} 1 & 0 & 0\\ 0 & 0 & 1 \end{array}\right]\left[\begin{array}{l} x\\ \dot{x}\\ \ddot{x} \end{array}\right]+\left[\begin{array}{l} z_{d}\\ z_{a} \end{array}\right]$$

where x, $\dot{x}$ and $\ddot{x}$ are the fused acceleration, velocity and displacement. $z_{d}$, $z_{a}$ and $z_{c}$ are the associated measurement noise of displacement and acceleration, and $x_{d}$ and ${\ddot{x}}_{a}$ are the measured displacement and acceleration. It is assumed that $z_{d}$, $z_{a}$ and $z_{c}$ are white noise Gaussian processes with covariance r and q, respectively.

Equations (3) and (4) can be compactly written in matrix form as:

(5) X=FX+Gw

(6) Z=HX+v

where F, G, and H are the measured system matrix, system noises matrix, and measurement matrix, respectively. v and w are the associated measurement noise of displacement and acceleration.

For the convenience of presentation, the measurement equation of displacement and acceleration is given separately:

(7) $$z_{d}=H_{d}X+Z_{d}$$

(8) $$z_{a}=H_{a}X+Z_{a}$$

where $z_{d}$ and $z_{a}$ are the displacement and acceleration measured value. $H_{d}$ and $H_{a}$ are the measurement matrix of displacement and acceleration.

Assuming the sampling period of the acceleration is $T_{a}$, the system equation and the observation equation are discrete as:

(9) $$X(k+1)=F_{d}X(k)+G_{d}w(k)$$

where $F_{d}$ and $G_{d}$ are derived by noting that $F_{d}$ is nilpotent (i.e., $A^{2}$ = 0)

(10) $$F_{d}=e^{AT_{a}}=\left[\begin{array}{ccc} 1 & T_{a} & T_{a}^{2}/2\\ 0 & 1 & T_{a}\\ 0 & 0 & 1 \end{array}\right]$$

(11) $$G_{d}=\int_{0}^{T_{a}}e^{Ff}Gd^{f}=\left[\begin{array}{c} T_{a}^{2}/2\\ T_{a}\\ 1 \end{array}\right]$$

(12) $$Q_{d}=\int_{0}^{T_{a}}e^{Ff}Qe^{F^{T}f}d^{f}=\left[\begin{array}{ccc} T_{a}^{5}/20 & T_{a}^{4}/8 & T_{a}^{3}/6\\ T_{a}^{4}/8 & T_{a}^{3}/3 & T_{a}^{2}/2\\ T_{a}^{3}/6 & T_{a}^{2}/2 & T_{a} \end{array}\right]e_{w}^{2}$$

(13) $$R_{d}=R_{Z_{d}}/T_{a}$$

(14) $$R_{a}=R_{Z_{a}}/T_{a}$$

The optimal state estimation of the state vector is obtained through multi-sensor Kalman filter.

Time update:

(15) $$\hat{x}(k+1|k)=F_{d}\hat{x}(k|k)$$

(16) $$P(k+1|k)=F_{d}P(k|k)F_{d}^{T}+Q_{d}$$

where $\hat{x}(k|k)$ and P(k|k) are a posterior estimate of the state vector and the system variance.

Measurement update:

(17) $$\hat{x}(k+1|k+1)=\hat{x}(k+1|k)+K_{d}(k+1)\left[z_{d}(k+1)-H_{d}\hat{x}(k+1|k)\right]$$

(18) $$P^{-1}(k+1|k+1)=P^{-1}(k+1|k)+H_{d}^{T}R_{d}^{-1}H_{d}+H_{a}^{T}R_{a}^{-1}H_{a}$$

The Kalman gain matrix of displacement and acceleration are given by:

(19) $$K_{d}(k)=P(k|k)H_{d}^{T}R_{d}^{-1}(k)$$

(20) $$K_{a}(k)=P(k|k)H_{a}^{T}R_{a}^{-1}(k)$$

In general, the displacement sensor and acceleration sensor have different sampling rates. In this paper, a fusion method based on multi-rate Kalman fusion is presented.

Suppose the sampling period of displacement is $T_{d}$, and it satisfies $T_{d}$/$T_{a}$ = M and M is a positive integer. Since there is no displacement measurement value in the $kT_{d}$ sampling interval, it can be approximated that the variance of the displacement is infinite when the Kalman filter data is fused, the $R_{d}$ approaches infinity, $K_{d}$ tends to zero, and only needed to time update and measurement update. The measurement update of the multi-rate fusion method is:

(21) $$\hat{x}(k+1|k+1)=\hat{x}(k+1|k)+K_{a}(k+1)\left[z_{a}(k+1)-H_{a}\hat{x}(k+1|k)\right]$$

(22) $$P^{-1}(k+1|k+1)=P^{-1}(k+1|k)+H_{a}^{T}R_{a}^{-1}H_{a}$$

It should be pointed out that, at the non-$kT_{d}$ moment, since there are no displacement measurements, only the acceleration time update and measurement update are performed. At the $kT_{d}$ moment, the acceleration and displacement measurements need to be updated simultaneously.

In order to improve the accuracy of the state estimation, the fusion results of multi-rate Kalman filtering need to be smoothed. Kalman filter smoothing is a linear combination of forward Kalman filtering and post-Kalman filtering state based on all observed values. According to the smooth way, there are three types, fixed-interval smoothing, fixed-point smoothing, and fixed-lag smoothing.

In this paper, the fixed-interval is firstly determined by combining the fixed-interval smoothing with fixed-interval smoothing. The state vector of the interval smoothing can be expressed as:

(23) $$\hat{x}(k|M)=\hat{x}(k|k)+A(k)\left[\hat{x}(k+1|M)-\hat{x}(k+1|k)\right]$$

(24) $$A(k)=P(k+1|k)F^{T}P^{-1}(k+1|k)....k=M-1,M-2,\cdots 0$$

where X(k|M) is the estimator for the smooth estimate of Kalman at k+1 step. A(k) is the smoothing gain matrix.

### 2.3. Correction of the Simulated Data through the Improved Multi-Rate Fusion Method

The selected signal is a swept-sine signal with an additional linear trend. The swept-sine signal is chosen because the exact analytical time-histories of displacement and velocity are known, so the errors can be calculated by subtracting the simulated signal from the fused signal. The performance of the improved fusion method can be evaluated by comparing the Root mean square error (RMSE) of Kalman fusion.

The time-history for the displacement can be expressed:

(25) x(t)=sin[(at+b)t]+ct

Through the first differentiation and the second differentiation, the velocity time-history and acceleration time-history can be respectively obtained:

(26) $$\dot{x}(t)=(2at+b)\cos[(at+b)t]+c$$

(27) $$\ddot{x}(t)=2a\cos[(at+b)t]-{\left(2at+b\right)}^{2}\sin[(at+b)t]$$

where a = $2\pi (f_{2}-f_{1})$/T and $f_{1}$ and $f_{2}$ are the start and end frequencies, respectively. T is the sampling time. c is the linear term coefficient.

It can be seen from Equations (26) and (27), that the linear drift term is undetectable in the acceleration term. Therefore, no matter how accurate the acceleration sensor is, the drift cannot be detected. The performance of the fusion can be analyzed and evaluated due to the real time expression of displacement and acceleration are known. Suppose the sampling frequency of acceleration is 1000 Hz and the sampling frequency of displacement is 100 Hz, so the ratio M is 10. Noise is added into the true values of displacement and acceleration. The remaining example uses the same method to load noise, which is Gaussian white noise with 10% Root mean square (RMS) noise-to-signal ratio. These noise levels are reasonable for civil engineering applications.

**Figure 1:**
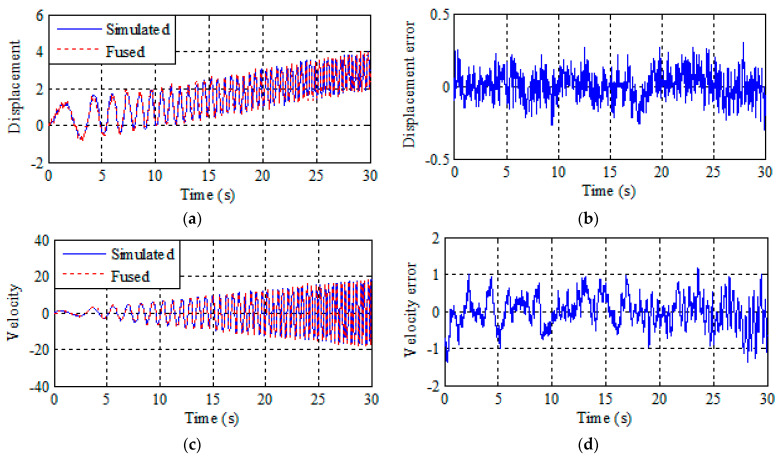
Traditional multi-rate fusion and estimation error of displacement and velocity. (**a**) Displacement traditional multi-rate fusion. (**b**) Displacement error. (**c**) Velocity traditional multi-rate fusion. (**d**) Velocity error.

**Figure 2:**
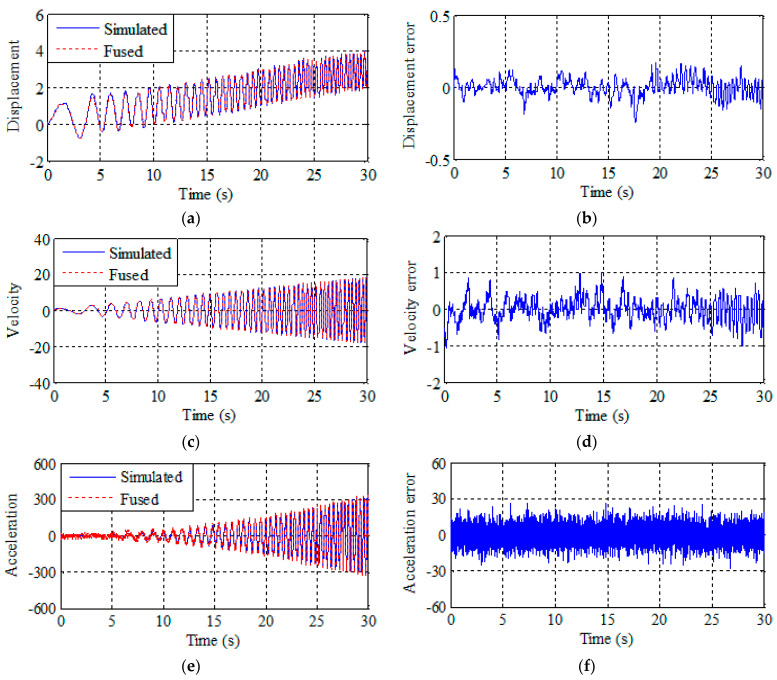
Improved multi-rate fusion and estimation error of displacement, velocity, and acceleration. (**a**) Displacement improved multi-rate fusion. (**b**) Displacement error. (**c**) Velocity improved multi-rate fusion. (**d**) Velocity error. (**e**) Acceleration improved multi-rate fusion. (**f**) Acceleration error.

The results of multi-rate data fusion and Kalman smoothing are shown in Figure 1 and Figure 2. The figures on the left are the fusion result and the figures on the right are the fusion error. It can be seen from Figure 1 that the traditional fusion method can only output displacement and velocity data and the error is relatively large. It can be seen Figure 2 that this scheme provides a good estimation of displacement, velocity, and acceleration, including tracking of drift terms. Figure 2 shows that the fusion result error is smaller than traditional fusion, and data can be corrected more accurately by smoothing after the improved multi-rate fusion. Better results are obtained by a simple comparison of the results with the traditional multi-rate fusion.

Two conclusions can be drawn by comparing the data in Table 1. First, the improved multi-rate fusion method is more accurate than the traditional multi-rate fusion method. Second, the traditional multi-rate fusion can only get the displacement and velocity fusion result. However, actual displacement and acceleration monitoring data of bridge mid-span need to be revised simultaneously. Improved multi-rate fusion proposed in this paper can obtain the results of displacement, velocity, and acceleration fusion simultaneously by setting two Kalman gain coefficients.

## 3. Fusion of Bridge Monitoring Data

### 3.1. Bridge and Monitoring System

In this paper, the monitoring data were analyzed and obtained from the Anqing Yangtze River Bridge, which is a long-span cable-stayed bridge located in Anhui province. The span configuration of the bridge is 50.0 + 215.0 + 510.0 + 215.0 + 50.0 m and the height of the two towers is 184.8 m. The bridge being studied was built and opened to traffic in 2004. An structural health monitoring system containing different types of sensors was installed on the bridge to continuously acquire various monitoring data, such as temperature, wind speed, displacement, acceleration, and strain. In this paper, the monitoring data of wind speed, lateral displacement, and acceleration of the bridge are investigated. The Anqing Yangtze River Bridge was chosen because displacement and acceleration sensors were installed in the mid-span of the bridge to verify the effectiveness of the improved multi-rate fusion method.

This paper analyzes the wind speed and direction monitoring data throughout 2014. Since the wind speed and direction monitoring data change rapidly, this paper calculates the average wind speed (AWS) to analyze the static characteristics of the wind. The displacement and acceleration data of the bridge span are fused by the improved multi-rate fusion method. Firstly, Global Positioning System (GPS) sampling rate of monitoring data of the Anqing Yangtze River Bridge is 1 Hz and that of the accelerometer is 20 Hz. According to the measured acceleration and displacement monitoring data, the multi-rate fusion step length ratio M = 20 is set. Then, the displacement and acceleration monitoring data are fused by the improved multi-rate fusion method.

**Figure 3:**
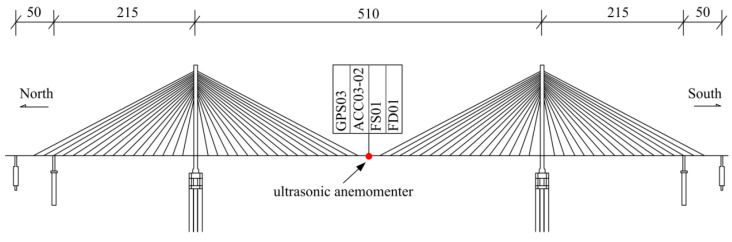
Elevation of the Anqing Yangtze River Bridge and sensor placement (unit: m).

The sensor and elevation of the Anqing Yangtze River Bridge involved in this paper are shown in Figure 3 and Table 2. As shown in Figure 3, the ultrasonic anemometer is installed at the middle of the mid-span girder. GPS and acceleration sensors are also installed at the middle of the mid-span girder. The detailed positions of the ultrasonic anemometer, accelerometer (ACC) and GPS sensors are presented in Figure 3. Moreover, the serial number and sampling frequency of the sensor are listed in Table 2.

### 3.2. Correction of the Measured Data through the Improved Multi-Rate Fusion Method

**Figure 4:**
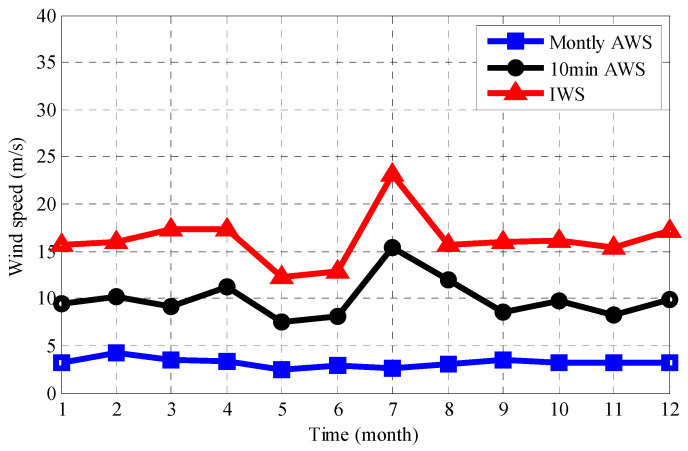
Variation tendency of the wind speed.

The 10-min average wind speed (10-min AWS) data are also calculated, which is usually used for correlation modeling with structural monitoring data. Figure 4 shows the AWS, 10-min AWS and maximum instantaneous wind speed (IWS) in each month of 2014. As shown in Figure 4, the variation range of the AWS is stable in the range of 2–4 m/s. As shown in Table 3 and Figure 4, the 10-min AWS has a large variation range, with the maximum value reaching 14.53 m/s. The 10-min AWS value was the highest in July and the lowest in May. The trend of the IWS is similar to that of the 10-min AWS. It can be seen from Figure 4 that the monthly IWS is always larger than the 10-min AWS, which can be attributed to the effects of fluctuating wind actions.

**Figure 5:**
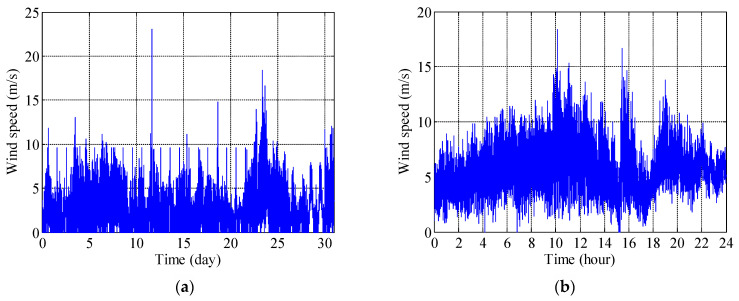
Wind speed–time curves for regular wind. (**a**) 1–31 July. (**b**) 24 July.

Considering the regular and relatively high AWS that occurred in July, the monitoring wind data on 6 and 24 July are analyzed to investigate the effects of the wind actions. Figure 5 shows the wind speed recorded by the structural health monitoring system for the whole month of July and 24 July. It can be seen from Figure 5a that the IWS in July reached 23.07 m/s, which was also the largest IWS in 2014. As shown in Figure 5a, the wind speed recorded by the monitoring system on 5, 8, 23, 24, and 31 July was relatively high. Figure 5b shows that the IWS on 24 July reached 18.4 m/s, which was much higher than the AWS of 8.8 m/s.

## 4. Accurate Modeling of Correlation between Lateral Wind Speed and Bridge Girder Displacement

Under strong wind action, the lateral wind speed in the mid-span of a bridge is the main influencing factor of displacement, which is positively correlated. Therefore, increasing the precision of lateral displacement in bridge span can effectively improve the precision of correlation modeling of wind speed and displacement. However, the sampling rate of speed and displacement is different in the actual monitoring data of a real bridge (Anqing Yangtze River Bridge). In this part, an improved multi-rate fusion method is used to fuse the displacement and acceleration data of the bridge mid-span, and then precise modeling and correlation analysis of the wind speed and fused displacement monitoring data are conducted.

The correlation between lateral displacement and wind speed in the bridge span is more significant under strong wind action. In this paper, the monitoring data of four periods of strong wind are selected for research. The monitoring data selected are as follows: the first monitoring data are from 01:00 to 10:00 on 5 July 2014 with the average lateral wind speed of 4.67 m/s. Monitoring data in the second section are from 12:00 to 20:00 on 8 July 2014 with the average lateral wind speed of 4.56 m/s. Monitoring data in the third section are from 12:00 to 18:00 on 23 July 2014 with the average lateral wind speed of 4.14 m/s. Monitoring data in the fourth section are from 09:00 to 16:00 on 24 July 2014 with the average lateral wind speed of 4.25 m/s. In this paper, the selected four cases under strong wind action are respectively defined as Case 1, Case 2, Case 3, and Case 4.

**Figure 6:**
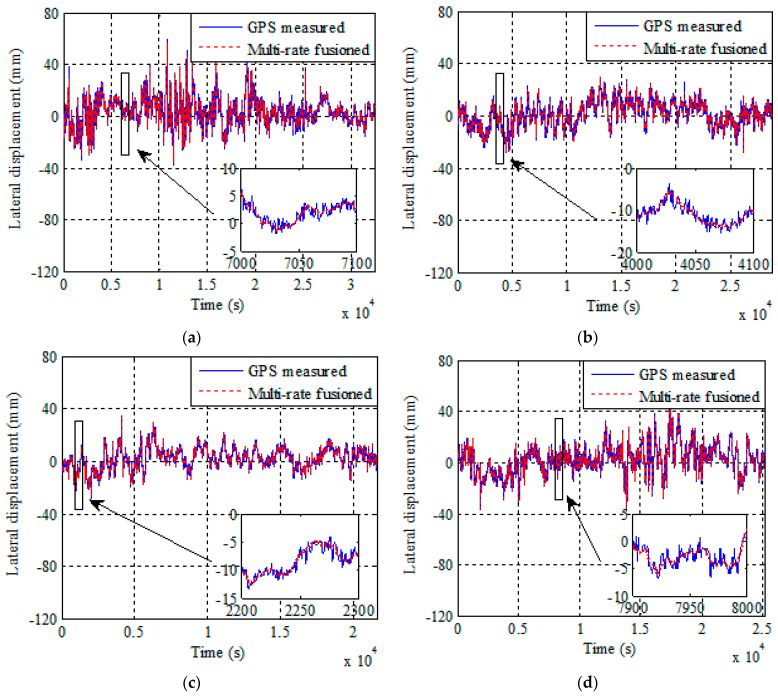
Global Positioning System (GPS) measured displacements and improved multi-rate fusion displacement results from Cases 1–4. (**a**) Case 1. (**b**) Case 2. (**c**) Case 3. (**d**) Case 4.

The improved multi-rate fusion method was utilized to fuse the displacement and acceleration monitoring data in the selected four cases under strong wind action. Figure 6 shows the results of displacement fusion in four cases before and after fusion. The solid line represents the displacement line before fusion, and the dashed line represents the displacement line after fusion. It can be seen that the displacement monitoring data are modified by the acceleration monitoring data to obtain more accurate displacement data in time domain. The enlarged images in the lower right corner of Figure 6a–d more clearly shows that the fusion method can eliminate noise from the displacement data.

**Figure 7:**
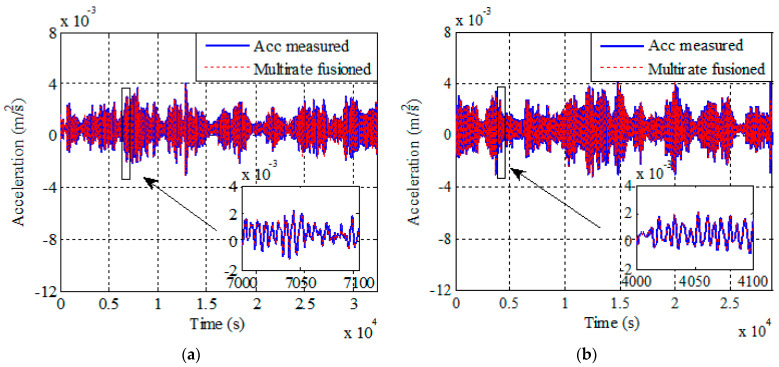

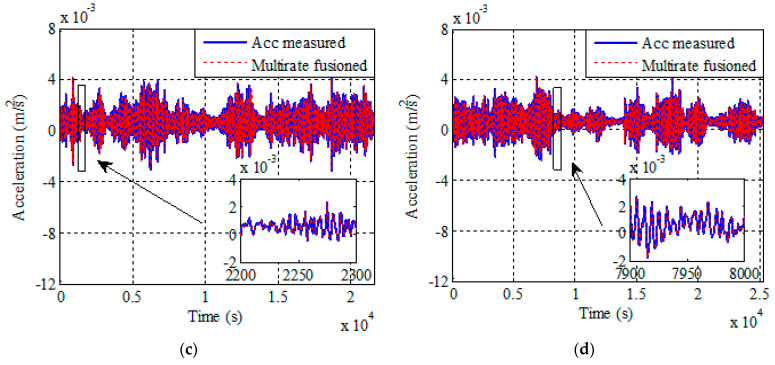
Acceleration measured data and improved multi-rate fusion acceleration results from Cases 1–4. (**a**) Case 1. (**b**) Case 2. (**c**) Case 3. (**d**) Case 4.

Compared to the traditional multi-rate fusion method, the improved multi-rate fusion method proposed in this paper can simultaneously output the correction result of acceleration when the displacement data are modified. Figure 7 shows the acceleration fusion results of corresponding displacement monitoring data. In the figure, the solid line represents the acceleration monitoring data before fusion, and the dashed line represents the acceleration monitoring data after fusion. It can be observed that the fusion method has corrected the acceleration in the time domain. In the field of bridge monitoring, the acceleration data obtained after fusion can be used to obtain more accurate results in system identification, damage identification, etc.

**Figure 8:**
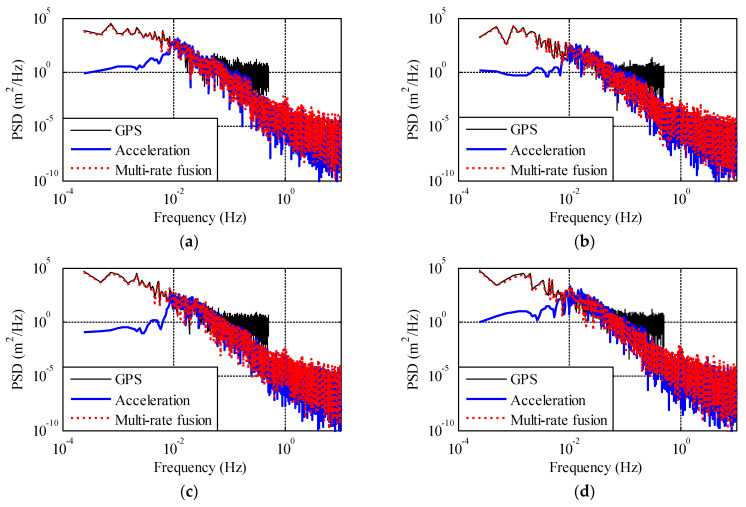
Power spectral density of GPS, ACC, and fused displacement from Cases 1–4. (**a**) Case 1. (**b**) Case 2. (**c**) Case 3. (**d**) Case 4.

Figure 8 is the frequency domain analysis results of the fusion of lateral displacement and acceleration in the mid-span of a bridge. The narrow solid line in the figure represents the Power Spectral Density (PSD) of the GPS and GPS sensor which has the disadvantage in high frequency stage. The bold solid line represents the PSD of the acceleration and acceleration sensor has disadvantage in low frequency stage. The dashed line is the PSD of the displacement date after fusion. It can be seen that the displacement after fusion is not only consistent with GPS information in the low frequency stage but also consistent with accelerometer information in the high frequency stage. Therefore, more accurate displacement data in the frequency domain is obtained by using the improved multi-rate fusion method.

### 4.1. Correlation Analysis of Lateral Wind Speed and Bridge Girder Displacement at Mid-Span

Generally, the influence of the wind load on the overall structure of the bridge is determined by wind tunnel test. The average lateral displacement of the main span of the bridge is positively correlated with the average lateral wind speed. In this paper, monitoring data are firstly fused and then the correlation modeling is carried out.

**Figure 9:**
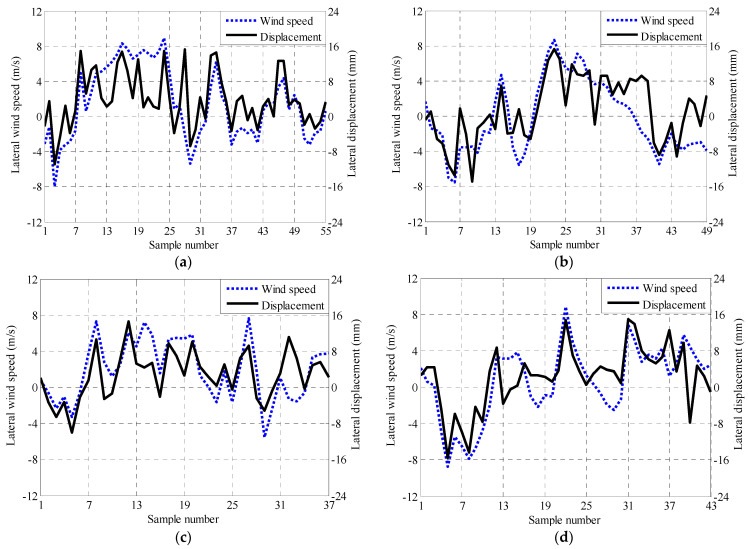
Relation diagram of lateral wind speed and displacement in the mid-span of the bridge after fusion from Cases 1–4. (**a**) Case 1. (**b**) Case 2. (**c**) Case 3. (**d**) Case 4.

Figure 9 shows the modeling results of lateral wind speed and displacement after fusion. The wind and displacement monitoring data of the selected cases under strong wind action were averaged in ten minutes. In the figure, the solid line represents the displacement monitoring data and the dashed line represents the wind speed monitoring data. It can be seen that the lateral displacement and wind speed in the span of the bridge are positively correlated and the correlation was strengthened in the analysis of correlation after fusion.

The correlation coefficient represents the correlation between the lateral wind speed and displacement in the mid-span of the bridge. In Case 1, 2, 3, and 4, the correlation coefficients of lateral displacement and wind speed before fusion were 0.686, 0.717, 0.641, and 0.715, respectively. After fusion, the correlation coefficients of lateral displacement and wind speed are 0.733, 0.759, 0.695, and 0.768, respectively. The correlation coefficient ranges from −1 to 1. The correlation coefficient after fusion of Case 4 in Table 4 is 0.768, indicating that the lateral displacement of the bridge is affected by the lateral wind speed under strong wind action. In addition, the correlation change can be explained more directly by the difference between the correlation coefficient of the fourth column and the change percentage of the fifth column. Table 4 shows that the more accurate modeling of the correlation between lateral wind speed and displacement is realized.

### 4.2. Regression Model of Lateral Wind Speed and Bridge Girder Displacement at Mid-Span

**Figure 10:**
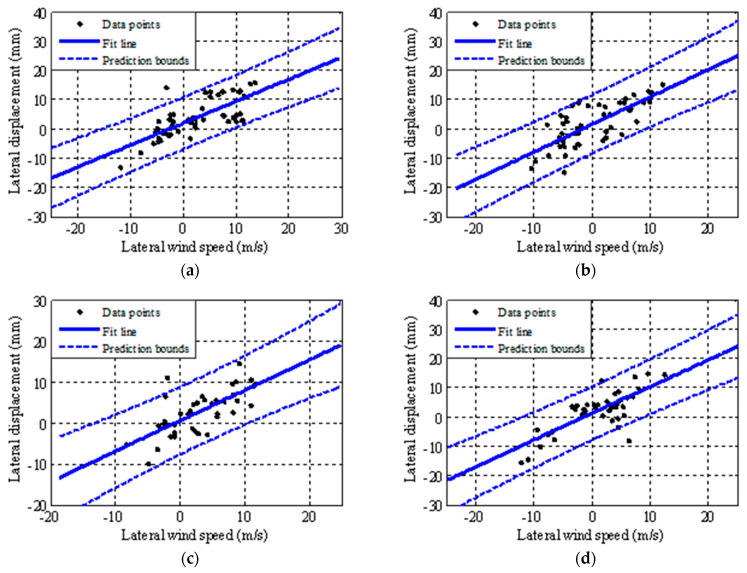
Linear regression model of lateral wind speed and displacement in the mid-span of the bridge after fusion from Cases 1–4. (**a**) Case 1. (**b**) Case 2. (**c**) Case 3. (**d**) Case 4.

The relationship between the lateral wind speed and the displacement in the mid-span of the bridge is studied by linear regression. The abscissa is the mean lateral wind speed of the bridge mid-span and the ordinate is the mean lateral displacement of the bridge mid-span. Linear regression is performed on the four cases selected in this paper, and the models before and after fusion are compared. Figure 10 shows the results of lateral wind speed and displacement regression model of the mid-span bridge after fusion. The regression model also reflects the increased correlation between lateral wind speed and displacement monitoring data modeling in the bridge mid-span.

Table 5 is the fitting equation of the regression model of lateral wind speed and displacement before and after fusion. Table 6 shows the change of confidence interval width before and after fusion under strong wind action from Case 1–4. The change in the width of the confidence interval is reflected more directly in the fourth and fifth columns. By comparing the second and third columns, it can be seen that the confidence interval width of the fitting equation decreases before and after fusion. Therefore, accurate modeling of lateral wind speed and displacement in bridge span are realized.

## 5. Warning Validity for the Performance Degradation of the Bridge Main Girder

When the bridge has performance degradation, the corresponding displacement response of the mid-span will increase. Therefore, this paper simulates the damage of the structure by increasing the displacement in the testing phase. The displacement corresponding to the structural damage is expressed by:

(28) $$S_{de}=S-\Delta$$

where S is the actual displacement of the bridge; Δ is the degradation of the displacement; and $S_{de}$ is the simulated value of the displacement after damage.

In this paper, seven performance degradation cases are set to verify the warning ability of the warning method. Equation (28) is used to simulate the seven performance degradation at the mid-span, north tower, and south tower. Table 7 shows seven detailed degradation degrees of the structure. In Table 7, Case 1 is the normal state of the structure, and the degradation degree of the structure increases successively from Case 2 to 7.

Three significance levels of 0.05, 0.01, and 0.003 were set to study the effects of the significance levels on the bridge performance warning. Before fusion and after fusion were applied to the actual monitoring data. The warning rate was the percentage of the warning sample number and the testing sample number. Finally, the performance warning of monitoring data was studied. Before fusion and after fusion are compared by using the warning rate as an index to evaluate the warning capability.

The warning rates of different damage cases for the mid-span are given in Table 8, respectively. It can be concluded from these tables that: (1) the larger the significance level is, the more likely the performance warning is to occur; (2) the warning rate of the fused data is higher than that of the data before fusion; (3) the warning rate of after fusion reaches 100% in case 7 at the mid-span, indicating that more than 30 mm of the damage can be detected.

**Figure 11:**
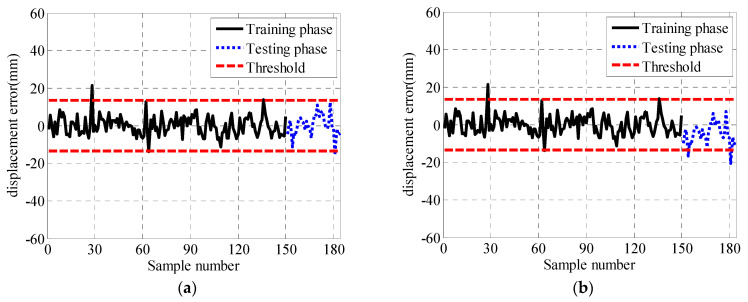

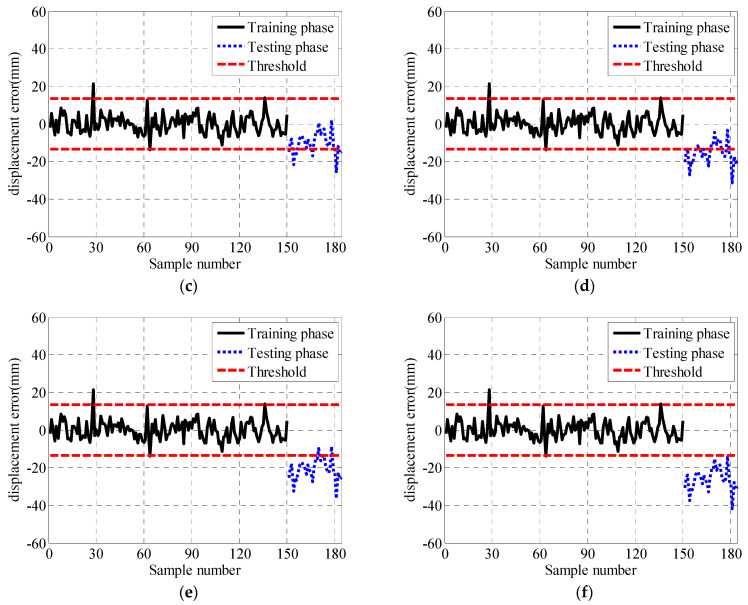
Warning results of the Shewhart control chart. (**a**) Case 1. (**b**) Case 2. (**c**) Case 3. (**d**) Case 4. (**e**) Case 5. (**f**) Case 6.

Figure 11 show the performance warning results of the statistics of warning rates after fusion in the mid-span of the bridge, where the significance level is 0.01. Figure 11a shows that Case 1 is the normal state of the structure. It can be seen from Figure 11b–e that compared with the training phase, the displacement errors of Case 2 to 5 in the testing phase have been more and more deviated from the center line of the control chart, and the warning number exceeding the threshold is also increasing gradually. It can be seen from Figure 11f that all test data in Case 6 exceed the threshold and the warning rate reached 100%. It is shown that the fusion method improves the modeling accuracy and can detect 25 mm damage in Case 6.

## 6. Conclusions

In this paper, the monitoring data of a cable-stayed bridge under strong wind action were investigated to reveal the relationship between the lateral wind speed and displacement. The traditional multi-rate fusion method is improved and then the displacement and acceleration monitoring data are modified by the improved method. More accurate displacement and acceleration monitoring data in time domain and frequency domain are obtained by using the improved multi-rate fusion method. Then the correlation between the lateral wind speed and bridge girder displacement at mid-span is modeled accurately. The conclusions of this study are drawn as follows:

1. Two Kalman gain coefficients are introduced to improve the traditional multi-rate fusion method. Compared with the traditional multi-rate fusion method, the improved multi-rate fusion method improves the accuracy of simulation and monitoring data in the time domain. It can be seen from the power spectrum that the fused displacement is consistent with the GPS information in the low frequency stage and the accelerometer information in the high frequency stage, indicating that the fusion method can correct the data in the frequency domain.
2. The traditional multi-rate fusion method can only correct the displacement data. The improved multi-rate fusion method proposed in this paper can modify the displacement and acceleration data simultaneously, which can be better applied to the monitoring data of the bridge than the traditional method. The accuracy of the fused displacement and acceleration data in time domain and frequency domain is improved.
3. Correlation modeling with the fused displacement data can significantly improve the modeling effect, increase the correlation coefficient, reduce the confidence interval width of the linear regression model, and, thus, achieve accurate correlation modeling between wind speed and displacement.
4. The performance warning of a cable-stayed bridge under strong wind is studied, and the capability of fusion method is verified. The warning rate of the fused data is higher than that of the data before fusion, and displacement damage with a severity of 30 mm occurring at the bridge main girder can be successfully detected.

## Figures and Tables

**Table 1:** Root mean square error (RMSE) of improved multi-rate Kalman fusion and smoothing results.

Methods	Measured Value	Multi-Rate Fusion	Smoothed
Traditional multi-rate fusion	Displacement	0.193	0.142
	Velocity	0.517	0.418
Improved multi-rate fusion	Displacement	0.112	0.062
	Velocity	0.354	0.257
	Acceleration	8.766	7.397

**Table 2:** Sensor displacement and specification.

Monitoring Subject	Position	Serial Number	Sampling Frequency (Hz)	Unit
Wind speed	Mid-span	FS01	1	m/s
Wind direction	Mid-span	FD01	1	Degree
Displacement	Mid-span	GPS03	1	mm
Acceleration	Mid-span	ACC03-02	20	m/s^2^

**Table 3:** Characteristics of the monthly wind speed in 2014.

Month	Average Wind Speed (m/s)	Maximum Instantaneous Wind Speed (m/s)	10-Min Average Wind Speed (m/s)
1	3.16	15.66	9.42
2	4.19	16.01	10.26
3	3.52	17.39	9.11
4	3.37	17.34	11.16
5	2.48	12.34	7.50
6	2.97	12.88	8.08
7	2.60	23.07	15.35
8	3.05	15.75	11.97
9	3.45	15.94	8.55
10	3.19	16.09	9.72
11	3.20	15.44	8.23
12	3.17	17.15	9.95

**Table 4:** Correlation coefficient of lateral displacement and wind speed in the bridge mid-span before and after fusion.

Case Number	Correlation Coefficient		Difference Value	Percentage Increase
	Before Fusion	After Fusion		
Case 1	0.686	0.733	0.047	6.81%
Case 2	0.717	0.759	0.042	5.87%
Case 3	0.641	0.695	0.054	8.40%
Case 4	0.715	0.768	0.053	7.37%

**Table 5:** Fitting equation of lateral displacement and wind speed in the mid-span of the bridge before and after the fusion.

Case Number	Fitting Equations	
	Before Fusion	After Fusion
Case 1	D = 1.029 V + 2.282	D = 1.128 V + 2.042
Case 2	D = 1.233 V + 1.660	D = 1.313 V + 1.621
Case 3	D = 1.078 V + 0.509	D = 1.116 V + 0.599
Case 4	D = 1.225 V + 1.595	D = 1.282 V + 1.349

**Table 6:** Confidence interval width of the fitting equation of lateral displacement and wind speed in the mid-span of the bridge before and after the fusion.

Case Number	Width of Confidence Interval		Difference Value	Percentage Decrease
	Before Fusion	After Fusion		
Case 1	0.601	0.568	0.033	9.01%
	2.629	2.396	0.233	9.73%
Case 2	0.683	0.635	0.048	8.52%
	2.802	2.621	0.181	7.16%
Case 3	0.885	0.843	0.042	7.70%
	3.331	3.078	0.253	8.22%
Case 4	0.733	0.690	0.043	8.88%
	2.978	2.784	0.197	7.09%

**Table 7:** Performance warning cases of bridge at the girder.

Case Number	Before Fusion (mm)	After Fusion (mm)
Case 1	ε = 0	ε = 0
Case 2	ε = 5	ε = 5
Case 3	ε = 10	ε = 10
Case 4	ε = 15	ε = 15
Case 5	ε = 20	ε = 20
Case 6	ε = 25	ε = 25
Case 7	ε = 30	ε = 30

**Table 8:** Warning rates for the performance degradation that occurred in the mid-span.

Case Number	Before Fusion (%)			After Fusion (%)		
	*α* = 0.05	*α* = 0.01	*α* = 0.003	*α* = 0.05	*α* = 0.01	*α* = 0.003
Case 1	2.94	0	0	2.94	2.94	2.94
Case 2	2.94	2.94	2.94	5.88	5.88	5.88
Case 3	5.88	5.88	5.88	29.41	23.53	14.71
Case 4	23.53	23.53	17.65	76.47	61.76	52.94
Case 5	70.59	50	50	94.12	91.18	88.24
Case 6	91.18	88.24	82.35	100	100	97.06
Case 7	100	97.06	97.06	100	100	100

## Data Availability

Data sharing not applicable.
